# Mothers in same‐sex relationships—Striving for equal parenthood: A grounded theory study

**DOI:** 10.1111/jocn.14971

**Published:** 2019-07-10

**Authors:** Heléne Appelgren Engström, Elisabet Häggström‐Nordin, Catrin Borneskog, Anna‐Lena Almqvist

**Affiliations:** ^1^ School of Health, Care and Social Welfare Mälardalen University Västerås Sweden; ^2^ Department of Medical and Health Science Linköping University Linköping Sweden; ^3^ Faculty of Health and Life Sciences Northumbria University Newcastle upon Tyne UK; ^4^ School of Health, Care and Social Welfare Mälardalen University Eskilstuna Sweden

**Keywords:** caring, encounters, parental support, parenthood, qualitative study, Sweden, two‐mother families

## Abstract

**Aims and objectives:**

To get a deeper understanding of how mothers in same‐sex relationships think and reason about their parenthood in terms of gender equality, and how they experience early parental support from child healthcare professionals.

**Background:**

There is an increasing amount of research on how women in same‐sex relationships experience healthcare services when forming a family. Yet there is limited knowledge of what kind of early parental support these women may request.

**Design:**

Grounded theory. Follows guidelines for qualitative research (COREQ).

**Method:**

Twenty women ranging from 25 to 42 years of age participated in semi‐structured interviews. Data collection and analysis took place in parallel, as recommended in grounded theory methodology.

**Results:**

The results are described by the core category *Same‐sex mothers request professional support to achieve equal parenthood*, which includes five categories: (a) equality in everyday life, (b) diversity in mother and child attachment, (c) justification of the family structure, (d) ambivalent thoughts about their child's future and (e) a special need for networking and request for professional support. These findings provide a deeper understanding of how same‐sex mothers experience their parenthood and the parental support that is offered.

**Conclusion:**

Child healthcare professionals need to be sensitive and recognise both mothers as equal parents and offer early parenting groups where two‐mother families feel included and supported.

**Relevance to clinical practice:**

Healthcare professionals need to be aware of diverse family formations and meet each parent as a unique individual without heteronormative assumptions. Same‐sex mothers must be treated as equal parents and acknowledged as mothers. Healthcare professionals should offer inclusive and supportive parental groups to same‐sex families. They should also inform and support nonbirth mothers about the possibility to breastfeed.

**What does this paper contribute to the wider global clinical community?**

- Same‐sex mothers’ experiences of parenthood and parental support from professionals at child health care.
- Recommendations to improve encounters and parental support at child health care.

## 1 INTRODUCTION

Since 2005, same‐sex mothers planning to start a family are offered assisted fertilisation with donated semen within the Swedish National Health Care coverage (Socialstyrelsen, 2005), and both mothers then become legal parents to their child (Riksdag, 2015). Previous research describes same‐sex mothers’ process of forming a family as stressful and with different decision‐making about donor and whom to be birth mother (Appelgren Engström, Häggström‐Nordin, Borneskog, & Almqvist 2018; Goldberg, 2006). In Sweden, one way of supporting parents is to invite them to take part in antenatal parental groups. Even though almost all parents are invited, only 40% participate (Lefèvre, Lundqvist, Drevenhorn, & Hallström, 2016). Parents describe that parental groups are of importance both to establish a network and to gain information during the child´s first year (Hjälmhult, Glavin, Okland, & Tveiten, 2014). Findings in a literature review show that parental support for same‐sex mothers in the Nordic countries is experienced as generally positive but heteronormative, meaning that heterosexuality is taken for granted (Wells & Lang, 2016). International studies also show that same‐sex mothers mostly have positive experiences, even though some experience of discrimination occurred when seeking health care for their children (Shields et al., 2012). As professionals at child health care have a responsibility to support families, it is important to understand same‐sex mothers’ experiences of parenthood and parental support from child healthcare professionals.

## 2 BACKGROUND

Both international and Swedish studies have shown that two‐mother families experience heteronormativity and stigma in their encounters with healthcare professionals (Andersen, Moberg, & Bengtsson Tops, 2017; Crouch, McNair, & Waters, 2017; Erlandsson, Linder, & Häggström‐Nordin, 2010; Malmquist & Zetterqvist Nelson, 2014; O’Niell, Hamer, & Dixon, 2013; Röndahl, Bruhner, & Lindhe, 2009; Wojnar & Katzenmayer, 2014). Despite the experienced heteronormativity, Swedish research show a satisfaction in same‐sex mothers relationship quality, few symptoms of anxiety and depression, and low levels of parental stress compared to heterosexual couples (Borneskog, Lampic, Sydsjö, Bladh, & Skoog Svanberg, 2014; Borneskog, Sydsjö, Lampic, Bladh, & Skoog Svanberg, 2013). Lesbian families described their emotional motherhood as mostly positive (Van Ewyk & Kruger, 2016).

Sweden has a generous paid parental leave, with 480 paid days off to be divided between the parents (Riksdag, 2015). As a consequence of this, equality among Swedish heterosexual parents is today more pronounced than among previous generations of parents, as parents now share parenting and parental leave more equally (Almqvist & Duvander, 2014). In same‐sex couples, some women describe equal parental roles as arising spontaneously, while others describe having to struggle for equality, or having unequal roles (Malmquist, 2015). In a study with same‐sex couples from USA, Downing and Goldberg (2011), highlighted that the mothers might both challenge and enact heteronormative constructions.

International and Swedish studies have reported how same‐sex mothers’ encounters with healthcare professionals were perceived as mainly satisfactory, though heteronormative. Moreover, nonbirth mothers report having a great and pressing need to be included in antenatal care and counselling, and also to be recognised as a mother and a parent to their child (Brennan & Sell, 2014; Dahl & Malterud, 2015; Erlandsson et al., 2010). Although the research on planned two‐mother families has increased, there is still limited knowledge about what kind of early parental support these mothers may desire and need. Therefore, our aim with this study was to gain a deeper understanding of how mothers in same‐sex relationships think and reason about their parenthood in terms of gender equality, and how they experience early parental support from child healthcare professionals. How do birthmothers and nonbirth mothers experience equal parenthood? How do birthmothers and nonbirth mothers experience early parental support from professionals at child health care?

## 3 METHOD

### 3.1 Design

To gain a deeper understanding of how mothers in two‐mother families think and reason about parenthood in terms of equality, and how they experience early parental support from healthcare professionals, we chose a qualitative approach. This paper provides conceptual description using grounded theory processes. Grounded theory (GT) is useful when exploring a relatively unknown area and is suitable for generating explanations of social issues (Corbin & Strauss, 2015). Corbin and Strauss’ (2015) description of GT was the choice as their approach to GT provide a clear structure for the analysis.

### 3.2 Recruitment of participants

Our inclusion criteria were as follows: birth mothers and nonbirth mothers in a same‐sex relationship having conceived via donation treatment at a Swedish clinic; children around 1–3 years of age; and parents having joint custody and living in central Sweden. Nurses at child healthcare centres distributed an informational letter to prospective participants, and the letter was also shared via a web page for lesbian, gay, bisexual, transgender and queer (LGBTQ) families. Those interested in participating then contacted the first author for further information. To get a maximum of diversity, we recruited participants from both rural and urban areas. Theoretical sampling is concept driven, meaning that the interviewer collected, coded and analysed data in an ongoing process until all categories were saturated (*n* = 20). The interviews took place during 2015–2016. Twenty mothers; eight couples (included eight birthmothers, eight nonbirth mothers) plus four separate birthmothers with variation in age and education participated. For demographics, see Table 1.

**Table 1:** Demographic characteristics of participants

Mothers	20
Birth mothers	12
Nonbirth mothers	8
Age (y mean 34)	
25–29	4
30–34	5
35–39	7
40–45	4
Number of children	
1 child	12
2 or more children	8
Marital status	
Married	13
Cohabiting	7
Length of relationship (mean 8.5)	
<5	2
5–10	13
>10	5
Educational level	
High school degree	6
University degree	14
Income per month before tax	
20,000 SEK	2
20,001–30,000	8
30,001–40,000	8
40,001–50,000	1
50,000	1
Type of housing	
Rented apartment	9
Owned apartment	4
House	7

### 3.3 Procedure and data analysis

We constructed an interview guide with open‐ended questions originating from the research questions and comprising themes as (a) planning for parenthood and (b) parental support. The first author tested the interview guide in a pilot‐interview with one birth mother and one nonbirth mother. No changes were made in the interview guide as a result, and these interviews were therefore included in the analysis, since they matched the inclusion criteria. The first author conducted each interview at a place chosen by the participant. The participants were given oral and written information, and also had the opportunity to ask questions about the study. Further, they gave written informed consent to participate in the study and filled out a form with demographic information. The interview then started with an open‐ended question: *Please tell me your thoughts about same‐sex parenthood.* The longer the process continued, the more detailed the questions became. The interviews lasted around 35 to 70 min and were recorded and transcribed verbatim*.* This data collection and coding were performed in parallel, as is characteristic of GT (Corbin & Strauss, 2015). The analysis strategy was constant comparative analysis and consisted of three steps, *open, axial* and *selective coding* (Corbin & Strauss, 2015). After each interview was transcribed, the analysis began with *open coding*, reading the material line by line to find codes characterising important information. In the second step, *axial coding* or coding around each category was conducted to determine its properties and the relationships between categories and subcategories. An example of the analysis process is described in Table 2.

**Table 2:** Examples of quotations, open codes, subcategories and categories emerging from the interviews

Quotations	Open code	Subcategories	Categories
We have divided it entirely the same … it is important that you both get to know everyday life with the children, it is super, otherwise you might not understand one another.	Shared parental leave	Sharing parental leave equally	Equality in everyday life
Me and my wife have thought of everything and talked about everything and equality is very important for us, therefore we are especially thankful that we both have been pregnant… it has been good for equality in our family	Both pregnant, good for equality	Sharing parental tasks equally	
It is important to be called mom, many ask us who is mother to the child?	Who is mother	Acknowledging both mothers	Justification of the family structure
Remove the word partner… I'm not even a partner to X, I'm her spouse, so to speak, legally speaking so for me it is a fact that my relation to the child has never been mentioned, never…	Relation to child not mentioned	Reluctance to be called a “partner”	
If I come by myself to the child health care, they almost always ask about the father, and not about the other parent… they always assume it is a man and a woman	Asking about the father	The sperm donor is only a donor—not a father	
She (the child) sees that other kids have a daddy and a mum and then comes the questions, then I think you might or ought to meet other families just for the sake of the child, and perhaps there is another family somewhere, where the children ask the same questions and want to meet other children	Meeting other two‐mother families	Networking with same‐sex parents	A special need for networking and request for professional support
Perhaps you can get some more help from child health care, how to talk to children about the donor, or that you can start a network	More help from child health care	Professional support from child health care	

S*elective coding* was the final coding step, aiming at reaching saturation of subcategories and categories, after which the core category was identified: *Same‐sex mothers request professional support to achieve equal parenthood*. According to GT, the core category should describe the essence of the study, and all categories should be related and linked to the core category (Corbin & Strauss, 2015). The first author wrote memos and diagrams throughout the entire process, and these were used to link and verify analytical interpretations with the empirical data. In the result's section, participants are mostly described as mothers, but separated as birth mother and nonbirth mother when relevant. When quoted, the mother's names have been replaced by pseudonyms. The material was divided and analysed in two parts: (a) the first part relates to the process of getting pregnant and support from antenatal care (authors published elsewhere) and (b) the second part describe experience of parenthood in terms of gender equality, and parental support from child healthcare professionals.

This paper follows guidelines for qualitative research (COREQ), see Supplementary File 1.

### 3.4 Ethical considerations

The Ethical Review Board in Uppsala, Sweden, approved this study (Dnr.2014/514). We gave the participants both oral and written information, and they were guaranteed confidentiality. We also informed them that participation was voluntary and that they could withdraw at any time without giving a reason. The mothers signed a declaration of consent before participating in the study.

## 4 RESULTS

The result describes twenty same‐sex mothers’ experiences of equal parenthood and parental support from child healthcare professionals. First, the core category will be presented, as it represents the main theme of the mothers´ experiences. Thereafter, the five categories that are linked to the core category.

### 4.1 The core category

**Figure 1:**
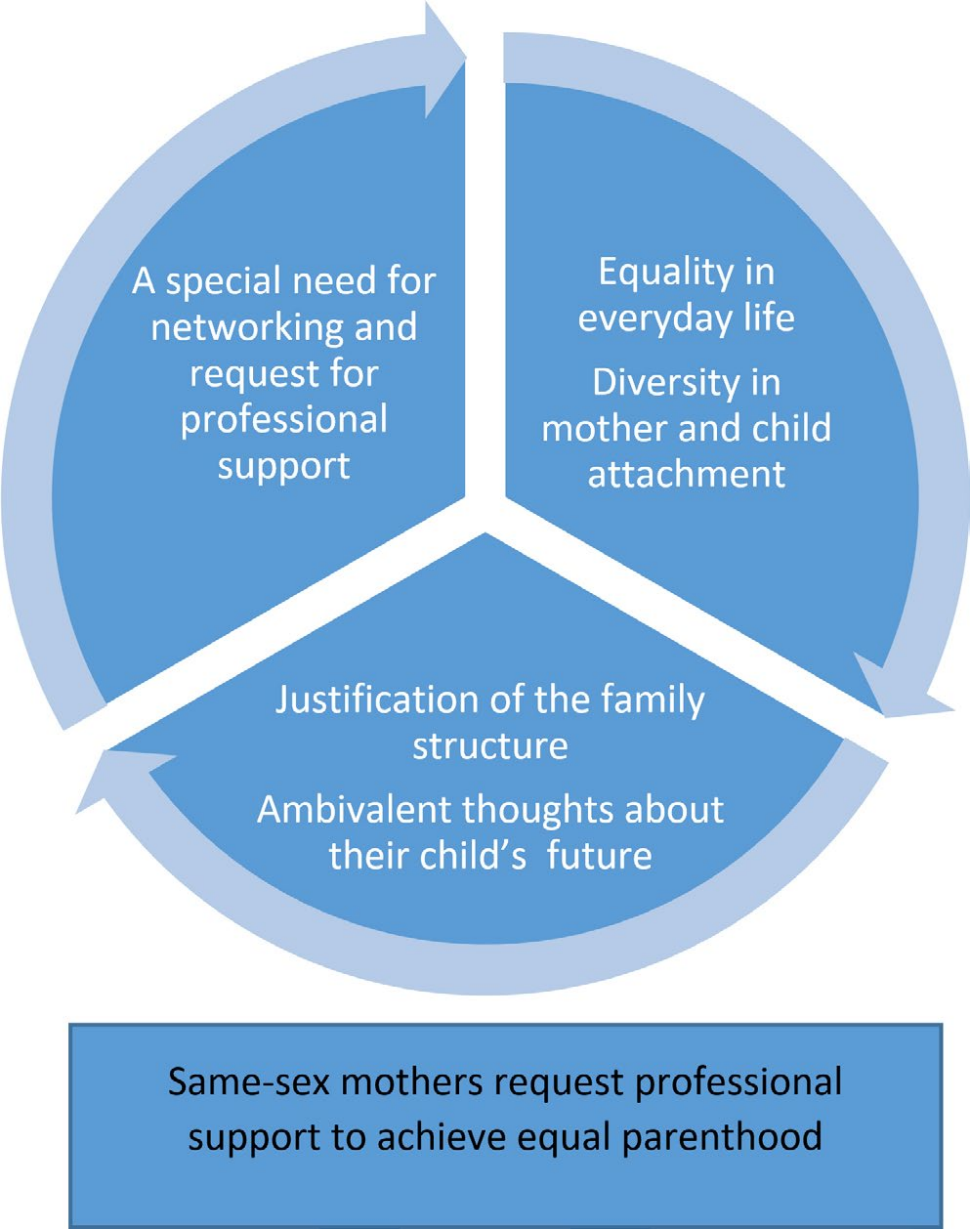
Parenthood and parental support from professionals, as experienced by mothers in same‐sex relationships

The core category, *Same‐sex mothers request professional support to achieve equal parenthood*, describes same‐sex mothers’ experiences of parenthood in terms of equality and early parental support from child healthcare professionals. The mothers expressed how they, within the couple, strived for equal parenthood in everyday life and in mother and child attachment. In encounter with healthcare professionals, they sometimes felt that they had to justify their family structure. The mothers struggled with ambivalent thoughts of hopes and fears, for their child´s future due to their own experience of breaking norms. Based on the mothers' experiences of striving for equal parenthood within the couple and the need to justify their family structure they had a special urge for networking with other same‐sex parents and a request for professional support. The core category includes five categories: (a) Equality in everyday life*,* (b) Diversity in mother and child attachment, (c) Justification of the family structure, (d) Ambivalent thoughts about their child´s future and (e) A special need for networking and request for professional support (see Figure 1). The first two categories, “Equality in everyday life” and “Diversity in mother and child attachment”, describe the mothers’ striving for equal parenthood within the couple. The following two categories, “Justification of the family structure” and “Ambivalent thoughts about their child's future”, describe the experience of encounters with healthcare professionals and thoughts about the surrounding society. The last category, “A special need for networking and request for professional support”, describes the importance of networking with other same‐sex families and individualised professional parental support.

#### 4.1.1 Equality in everyday life

The mothers strived for a high level of equality in their everyday life. They talked about equal parenthood as, sharing parental leave equally and sharing parental tasks equally.

##### Sharing parental leave equally

Most mothers described dividing the parental leave completely equally or at least having had that ambition. The mothers emphasized that it was important to share parental leave equally. As one mother expressed it: “We have divided it entirely the same … it is important that you both get to know everyday life with the children, it is super, otherwise you might not understand one another” (Ebba)*.* Some mothers described sharing equally because both of them wanted to spend time at home with their child: “We have taken the same amount of leave …. as we both want to be at home” (Ylva). Several mothers described that both mothers stayed at home during the first weeks or even month of their child's life. One mother said: “It went without saying that we would share the parental leave, both of us were at home for the first six months” (Frida). The mother who was not on parental leave often worked part‐time. Some mothers described having saved money to be able to stay at home with their long‐awaited child. Others said that both mothers wanted to stay home when their child was ill. The mothers described sharing parental leave equally as a matter of fairness, because both mothers wanted to spend as much time as possible at home with the child. Sharing parental leave equally was also an important way to show that both mothers and their professional lives were just as important. Often, the birthmothers stayed at home with the child for the first few months, for practical reasons such as breastfeeding. The participants expressed that some people had quite a lot of opinions about how the mothers shared their parental leave. One nonbirth mother's experience when applying for parental leave was as follows: “There is an expectation that I should stay home for a somewhat shorter time than I requested” (Jenny). A few birthmothers and nonbirth mothers said that the birthmother took almost all the parental leave, but that they regretted this and planned to share the parental leave more equally the next time.

##### Sharing parental tasks equally

Almost all the mothers stated that their intention was to share parenting tasks equally and also to share all housework. The mothers also pointed to the importance of sharing household tasks equally: “I think we do everything together, we are equal” (Johanna). Some said that it might be easy to be equal because they have been raised in an egalitarian way: “It works incredibly painlessly, two who think, two in charge” (Ylva)*.* In couples where both mothers had experience of pregnancy, the parents were grateful for that fact and thought it benefitted the equality of their relationship. “Me and my wife have thought of everything and talked about everything, and equality is very important to us, therefore we are especially thankful that we both have been pregnant…. it has been good for the equality in our family” (Lotta), as one mother expressed it. It was good to have experience from “both sides”; she thought it contributed to their having a deeper understanding of each other*.* Some mothers said that they were equal, even though they did different things at home: “We are two women and we do everything together… I may do more classical female work while [her wife] is really good at repairing, and I am sewing and cooking food and stuff” (Vanja), suggesting that her family might look like a traditional family, with stereotypical gender roles.

#### 4.1.2 Diversity in mother and child attachment

The mothers described attachment to the child as arising and being expressed in different ways, such as through breastfeeding, by spending time together or by being physically close and cuddling.

##### Attachment through breastfeeding

Almost all mothers said that breastfeeding contributed to natural attachment. One birthmother said: “The child wanted to be with me, breastfeeding provides natural attachment”*.* Another birthmother said: “I think there will be a more natural attachment for those who are breastfeeding, in the beginning anyway”. The mothers thought that there was a difference in stages of attachment; when the child was an infant, it seemed to prefer to be with the mother who was breastfeeding. One nonbirth mother expressed that she thought the child loved her birthmother more while breastfeeding: “They [the kids] love her more, maybe not love but, it's so, when they were very young they wanted to be with her all the time. Mom, mom, mom, and she also nursed. Now I think she [the oldest girl] loves us both the same, because there's not a big difference” (Heidi). For some mothers, it was important that both could feed the baby: “We also introduced a nursing bottle, I used a breast pump so that [the nonbirth mother] could nurse with the bottle too, so we both fed her, and I think, I think it means a lot, that we've both done it” (Ann). Another nonbirth mother expressed that she could understand how fathers could feel a bit left out when the child was being breastfed. Just a few nonbirth mothers had knowledge about the possibility to breastfeed their child. One nonbirth mother had tried to breastfeed but it did not work. Another mother said that the nonbirth mother probably would have tried to breastfeed if they had been informed about the possibility and supported by healthcare professionals. She said: “We read articles about it, found something on the Internet that said you could take hormones or that it could work naturally to… felt taboo…don't know anybody that has done this… does not follow the norm…but with support, I think we would have tried” (Pia).

##### Attachment through spending time and being physically close and cuddling

The mothers talked about attachment developing by spending time with and being physically close to their child. “The time you spend with your child is important – not how you feed it” (Lotta)*.* One mother expressed that there was no reason to believe that the nonbirth mother would have difficulty becoming attached to the child, but at the same time she was worried that she [the nonbirth mother] had “forbidden thoughts” as she put it and wondered: “if she wishes that she would get to be pregnant someday or if she believed it would be different” (Ylva). Some mothers described that their child bonded to the birth mother through breastfeeding in the beginning. But as time went by, and the nonbirth mother spent more time with her child, she sometimes became the child's first choice. Mothers who had the experience of both being a birth mother and a nonbirth mother felt that it took longer to bond with the child she had not given birth to. One mother said*: *“I think it was harder to form an attachment to [the child she is nonbirth mother to], it took longer, it did. I didn't think it would be that way, but it took staying at home a lot….it's not easy to just get that relationship, you have to work for it” (Lilly). The mothers described that spending time with the child was of importance for forming attachment to the child.

#### 4.1.3 Justification of the family structure

Two‐mother families sometimes felt that they had to justify their family structure in encounters with healthcare professionals. Both birthmothers and nonbirth mothers expressed the importance of being acknowledged as mothers, were reluctant to be called a “partner” and highlighted that the sperm donor was just a donor ‐ not a father.

##### Acknowledging both mothers

The participants highlighted the necessity that both mothers are acknowledged and spoken to as mothers and equal parents. One nonbirth mother told that healthcare professionals asked what to call her. She said: “We say mother and mother because we are mother and mother” (Diana) and told that their daughter says: “mother [her name] and mother [partner's name]” (Diana). The participants felt supported in their parental role when healthcare professionals at child healthcare centres addressed them both as mothers. The mothers said they called themselves mothers and said it was important to be called mothers, or parents. “It is important to be called mom. Many ask us who is the mother of the child?” Being asked that question, “who is the mother of the child?” made the nonbirth mother feel excluded, unless the issue was essential from a biological perspective.

##### Reluctance to be called a “partner”

The term “partner” was perceived differently by the participants. Most mothers expressed that they did not want to be called a partner by the healthcare professionals, because they were not partners to their child. As one mother said: “I do not want to be called partner, I’m not a partner to my child, I am a mother too.” Another mother expressed: “Remove the word partner, I’m not even a partner to [her wife], I’m her spouse, so to speak, legally, my relationship to [her child] has never been mentioned, never” (Ella). This quotation shows that using the term partner did not validate this mother in her parental role. One mother, on the other hand, highlighted the benefits of using the term partner: “It's so easy just to say partner, you don't need to say dad or the other parent, you can just use the term partner, it doesn't matter what gender you are” (Pia).

##### The sperm donor is only a donor—not a father

Some mothers had not been asked about the donor or the father. Others had to face questions about the donor and “the father” from healthcare professionals. A few mothers described being asked about “the father” if they went by themselves to the child health centre. “If I went by myself to the child health center, they almost always asked about the father and not about the other parent… they always assumed there is a man and a woman” (Therese). The mothers had learned at the fertility clinic that they should talk to their child about the donor, but avoid referring to the donor as a father. When the mothers then went to the child health centre with their child, some were met by questions about the father. “At the child health center there has been different staff, we have met very many and there has almost consistently been something that we have had to raise; we are two mothers, there is no father, there is a donor…. We were often asked, even at the child health center, how is it, do you know what the father looks like?” (Pia). Being asked about “the father” was something the mothers did not appreciate, especially if their older children were also with them. Another mother described how healthcare professionals asked: “What do we know about the donor?” when discussing a child's suspected milk allergy. However, this mother did not want to talk about the donor or answer that kind of question, and she felt that healthcare professionals ought to know what information the mothers have about the donor.

#### 4.1.4 Ambivalent thoughts about their child's future

The mothers described their thoughts about the future in terms of hopes and fears for their child, based on their assumption that, like them, their child will face prejudice and ignorance due to its living in a non‐nuclear family. The mothers talked about how their family structure might affect the child's future.

##### Hopes for their child

Almost all mothers described the future in terms of hopes and fears. They hoped for a mentally strong child, or that they could raise their children to be mentally strong. The mothers believed that their child was always going to be regarded as different and would be asked about their family structure. The mothers talked about violating norms, from their own experience, and worried that their own decisions might affect the child's future. One mother expressed it as follows: “We will always be different, you don't need to put anything negative in it …. but I hope that she can be a strong person… she will always get questions and she will always be a bit different” (Christine). Some mothers expressed a belief and hope that society would change. One birth mother said: “But then I think society is getting more and more open, if you just look at the past 20 years, what has happened, there's a huge difference, so I hope it will continue…” (Malin). They hoped that in the future society would be more open and permissive, with an increased number of same‐sex families.

##### Fears about the future

The mothers feared the future because they thought their child would always be questioned and considered different. One birth mother of twins said: “Sure, you're still afraid… it's getting harder and harder to control the situation… thankfully they have each other, but it may not be easy to be the only family that breaks the norm, it's so horribly unfair that my own choice has put them in the family they're in…” (Frida). The mothers argued that their child had to bear the consequences of the mothers’ decision: “That makes me rather worried, I'm trying not to be, but I'm worried about how others, if he's going to get crap from other people because he has two moms” (Malin). Another mother expressed worries about the future: “There are some thoughts about the future, you don't want there… to be any reason for bullying, of course, or anything else that's bad, so I think we must try to be as open as possible, but it's not possible to control everything” (Frida). Another mother was thinking more and more about what the future would be like for her child as she grows older, that there probably would be more questions about why she did not have a dad like everyone else. Mothers in rural areas thought about the future and what it would be like for their children at school and with friends. As one birth mother said: “…a bit more worried about what it will be like for her, just because it's a small place, and everyone knows everything about everyone…” (Eva). Some mothers thought they might need to move for the sake of the children. One mother said that they avoided certain things and situations and that they did not travel to certain countries because it did not feel safe.

#### 4.1.5 A special need for networking and request for professional support

There was a special need for networking and a request for professional support among the mothers. The mothers talked about the importance of networking with other same‐sex families, both for their own sake and that of their child. They also highlighted their request for professional support from healthcare professionals.

##### Networking with same‐sex parents

The mothers expressed a need to network to meet other same‐sex families. Not for their own sake, but so their child could see that there were other children with two mothers. One mother said: “My oldest girl asks why she doesn't have a dad. Are there more children with two mothers? And why don't we meet these families?” (Vanja). The mothers thought networking would be good for their child, but not all participants had access to a network. They thought it would be good to have contact with other same‐sex parents. “We don't need support to live together, but I think it is mostly for [the child], so he can grow up and see that there are others too. I think we can tell him, but it will be clearer if he sees other children who also have two moms, or two dads for that matter, yes, I would like that” (Malin). The mothers expressed that it would be good to meet other same‐sex families, so that the child could see that there were other families like them. “She [the child] sees that other kids have a dad and a mom and then come the questions, then I think you might want to or ought to meet other families just for the sake of the child, and perhaps there's another family somewhere, where the children ask the same questions and want to meet other children” (Vanja). Some mothers also expressed that they had a need to network with other same‐sex families, not only for their child's sake but also for their own. These mothers emphasized the importance of meeting other same‐sex families to talk and exchange experiences about preschool and school matters. Some mothers had social networks with other two‐mother families. One mother expressed it as follows: “The network has been a help for us, it's good to know someone who has been through the same process” (Irene). Some mothers mentioned a “support‐group” on Facebook that was of importance for them.

##### Professional support from child health care

Most mothers felt that they received good support from healthcare professionals. One said: “… it was a great treat, it was like no wonder… our nurse [at the child health center] is absolutely amazing.” (Christine). However, not all participants had such good experiences. One mother mentioned that the child health centre did not have a parental group. Other mothers decided against attending parental groups because the group was called a mother‐and‐father group, which made the mothers feel that this was not directed to them. A few mothers experienced that there was a division into *us* and *them,* and therefore thought it would be good if there was an association for same‐sex parents. Some nonbirth mothers did not feel it was relevant for them when healthcare professionals talked about how the mothers’ bodies had changed after giving birth. The mothers had specific needs; as one mother expressed it: “Perhaps you can get some more help from child health care, how to talk to children about the donor, or you can start a network” (Malin). The mothers expressed a need to meet and socialize with other same‐sex parents and talked about a parental group for same‐sex parents. There was only one mother who had experience of parental groups for LGBTQ parents organised by child health care, which was something she appreciated a lot: “I think that the support for families at child health care is very good… we are not the only same‐sex family” (Lotta). A few nonbirth mothers reported that as nonbirth mothers they did not get the same support from healthcare professionals as the birth mothers did. One nonbirth mother requested support for the parent who does not give birth to the child: “to feel like the secondary parent, and not to be equally needed” (Ella). She also wondered whether she would get the same support as birthmothers get, for example in case of depression. For some nonbirth mothers, it felt like they had a strange “in‐between role”, occupying an intermediate position like a supporting character. One nonbirth mother experienced that healthcare professionals had unrealistic, traditional and stereotypical gender expectations: “…I would have a more natural approach to parenting than a man in the same situation…. it would come more naturally for me to take responsibility and be involved… I would be a better parent than a man…” (Jenny). But at the same time, nobody had asked her what it is like to be a nonbirth mother.

## 5 DISCUSSION

The present study aimed to achieve a deeper understanding of how mothers in same‐sex relationships think and reason about their parenthood, and how they experience early parental support from healthcare professionals. The core category—*Same‐sex mothers request professional support to achieve equal parenthood*—refers to the mothers’ stories about how they shared parenthood equally within their family. The lack of recognition of the nonbirth mother as an equal parent, and the mothers’ need to justify their family structure in encounters with healthcare professionals, made the mothers worry about their child's future. This led to a need for networking with other same‐sex parents and a request for individualised support from healthcare professionals.

The first category describes equality in everyday life. Mothers in two‐mother families aimed for a high level of equality in their relationship and parenting and also aimed high when it comes to sharing their parental leave equally. Similar results, where lesbian mothers idealized equal parenthood, are also described by Malmquist (2015). Equal parenting seemed to be easier to achieve within the family than in encounters with others. In meetings with other people, the mothers had to defend their family structure in the face of “norms”. The mothers talked about equal parenting despite differences in mother and child attachment.

The second category describes diversity in mother and child attachment. The mothers who breastfed said it gave them a feeling of natural attachment, and some of the mothers also expressed that when the child was an infant, it seemed to prefer the mother that breastfed. The same result, that infants prefer the birth mother while breastfeeding, was found in a study where lesbian mothers described their children's parental preferences (Goldberg, Downing, & Souck, 2008). Only a few mothers knew about the possibility for the nonbirth mother to try and breastfeed her child, and one mother felt that it was considered “taboo”. This feeling of taboo can be explained by the current norms and values in society.

The third category, justification of the family structure, highlights that the mothers were not always treated as equal parents in encounters with community services, such as child health centres. The mothers in this study expressed that both partners were mothers. However in encounters with healthcare professionals, the nonbirth mother sometimes felt excluded. This feeling of exclusion is an example of what Halldórsdóttir (1996) calls uncaring encounters, which are characterised by a lack of respect and genuine concern and not taking notice of the patient, in this case the nonbirth mother.

The participants were also asked questions about “the father”. This heteronormative view, where everyone is expected to be heterosexual (Connell & Pearse, 2015), seems to be especially prominent in child healthcare. Previous research has reported that two‐mother families often are met by heteronormative language (Andersen et al., 2017; Crouch et al., 2017; Erlandsson et al., 2010; Malmquist & Zetterqvist Nelson, 2014; O’Niell et al., 2013; Röndahl et al., 2009; Wojnar & Katzenmayer, 2014). In this study, some nonbirth mothers were called partners, but most nonbirth mothers in our study did not want to be called partners, as they were mothers too. Every family is unique, and healthcare professionals need to be very responsive to all parents to be able to use the “right” and appropriate words in communicating with families. Female couples have had access to assisted reproduction techniques (ART) at Swedish clinics since 2005 (Socialstyrelsen, 2005), but changes in the law still do not seem to have changed the content or organisation of child health care. Meeting with heteronormative assumptions from healthcare professionals might jeopardize parents’ trust in health care and willingness to participate in parental groups.

Some nonbirth mothers in this study felt like secondary parents, but felt validated when healthcare professionals addressed both parents as mothers. This might be one way to increase these mothers’ health and well‐being and empower them in their parenting role, as described by Halldórsdóttir (1996).

The fourth category is about the mothers’ ambivalent thoughts about their child´s future. Almost all the mothers talked about their child's future and were worried about how their two‐mother family structure could affect their child. This concern for the child, who has to live with its mothers’ choice, was also expressed by lesbian mothers in the USA (Wall, 2011). Some mothers in this study hoped that their child would be a mentally strong person and some feared their child would face difficulties. Recent research results about children in two‐mother families showed that these children were met by questions about their family structure (Raes et al., 2015). Despite this, children growing up in two‐mother families were shown to be well‐functioning in school (Rosenfeld, 2010).

The last category describes the mothers' experience of networks and support. A need for networking with other same‐sex parents was clearly highlighted by the mothers. Those who had access to a network of same‐sex families felt supported, but many mothers did not have access to a network. The importance of network with other same‐sex families is also described by Álvarez‐Bernardo and García‐Berbén (2018) but in their study most participants had access to a network. Maybe this need for networking is a result of mothers choosing not to join a parental group at the child health centre, or not even being offered to participate in a parental group. Previous research has reported that same‐sex parent groups are important for the children; meeting with other two‐mother families is a way to give the children other examples of this family form (Suter, Daas, & Bergen Mason, 2008). Early parental support is of importance for the well‐being of parents, although research has shown that nonbirth mothers felt marginalised and like a secondary parent in parental groups (Wells & Lang, 2016). Some nonbirth mothers in this study felt like a secondary parent and experienced both high expectations and a feeling of exclusion from healthcare professionals. The mothers’ request for support and need for networking indicates that nurses at child health centres should organise parent groups with a broader scope of parental inclusion than is the case today. This is especially important as previous research has found that lesbian women choosing motherhood sometimes lose the support of both their families and the lesbian community (Wall, 2011).

### 5.1 Strengths and limitations of the study

This study contributes to a deeper understanding of how mothers in a same‐sex relationship think and reason about their parenthood and how they experience early parental support from healthcare professionals. One strength of this study is the rich descriptions of participants’ experiences. Another one is the variation in participants’ age, education and geographical spread, which increase the transferability and credibility of the study (Lincoln & Guba, 1985). The first author did the interviews and transcriptions, the co‐authors read and coded some transcriptions, to ensure credibility and dependability (Lincoln & Guba, 1985). To ensure confirmability, the first author also discussed the categories and core category with the co‐authors, who are experienced in grounded theory. The four criteria credibility, dependability, confirmability and transferability were discussed to ensure trustworthiness (Lincoln & Guba, 1985). The findings contribute to the growing body of research on two‐mother families that conceived through sperm donation treatment at Swedish fertility clinics. One limitation could be that both first‐time mothers and mothers with more than one child participated, as this might affect both the experience of parenthood and support from child healthcare professionals. However, in spite of close readings, it has not been possible to see that this has affected the result. Another limitation is that this study presents a conceptual description, not a theory. Further research is needed to develop a theory.

## 6 CONCLUSION

The core category, *Same‐sex mothers request professional support to achieve equal parenthood,* describes how mothers in same‐sex relationships experience parenthood and early parental support. Equality was of importance in most of these two‐mother families, and the mothers described themselves as aiming for a high level of equality. They expressed a desire to be treated as a family, and as equally valid mothers, by professionals at child healthcare services. Using appropriate terms and vocabulary in communication is one way to support and strengthen the mothers in their parental role. Healthcare professionals should also support and advise nonbirth mothers who wish to breastfeed and offer parental groups designed to also welcome two‐mother families.

## 7 RELEVANCE TO CLINICAL PRACTICE

In this study, same‐sex mothers’ experiences of parental support from child healthcare professionals are described, and the results highlight how encounters and parental support can be improved. Healthcare professionals need to be aware of diverse family formations and meet each parent as a unique individual without heteronormative assumptions. One way to do this is to ask open questions and be sensitive to what concepts the parents use in their family. Same‐sex mothers must be treated as equal parents and acknowledged as mothers. Nonbirth mothers need to be strengthened and supported in their new role as a mother and also be informed and supported about the ability to breastfeed. Healthcare professionals should offer inclusive and supportive parental groups to same‐sex families. Parental groups have several important functions, first, to support the parents in the attachment process to their child and second to meet the parents' concerns about the child. If possible, we recommend to offer parental groups with other same‐sex parents or refer to a network for same‐sex parents, as this is something that is requested by the parents themselves.

## CONFLICT OF INTEREST

The authors have no conflicts of interests to declare.
